# Fundamental insights into gallium leaching for sustainable electronic waste recovery

**DOI:** 10.1038/s41598-025-30908-3

**Published:** 2025-12-02

**Authors:** Aylin Nur Erkmen, Roland Ulber, Thomas Jüstel, Mirjam Altendorfner

**Affiliations:** 1 The Department of Chemical Engineering, FH Münster University of Applied Sciences, Steinfurt, 48565 Germany; 2 The Department of Bioprocess Engineering, The University of Kaiserslautern-Landau, Kaiserslautern, 67663 Germany

**Keywords:** Critical metals, Gallium, Metal leaching, Oxalic acid, Sustainable chemistry, Chemistry, Environmental sciences

## Abstract

This study systematically concerns the leaching behavior and dissolution kinetics of gallium (Ga). The objective was to identify sustainable leaching agents by incorporating organic acid reagents. Using a novel approach involving pH-adjusted experiments, the findings elucidated the dissolution behavior, delineating acidolysis and complexolysis. The screening results demonstrated the efficacy of oxalic acid (10 mM), which extracted 1105.2 ± 61.4 mg/L of Ga at pH 1.2. Dissolution kinetics based on the shrinking core model (SCM) revealed a synergistic mechanism governed by film diffusion and surface chemical reaction control, with chemical reaction control dominating at higher temperatures. One-factor-at-a-time (OFAT) experiments clarified the influence of experimental parameters on Ga leaching yield. In addition, the Box-Behnken design (BBD) was employed to evaluate parameter interactions, confirming the significance of the process parameters, including interaction terms, while characterizing the model in terms of a quadratic expression. Under optimized conditions: 710.55 mM acid concentration, a reaction temperature of $84.5^\circ$C, and a solid loading of 50 g/L, 37% of Ga was effectively extracted within 3.2 hours. These findings underscore the selectivity and operational compatibility of oxalic acid compared to conventional leaching agents, emphasizing its promising integration into biogenic production pathways and sustainable closed-loop gallium recovery processes.

## Introduction

Gallium (Ga) has been classified as a critical metal by the European Commission owing to its indispensable role in semiconductors, photovoltaics, displays, wearable devices, and other advanced technologies, underscoring its importance considering the surge in demand centering on this element^1,2^. Gallium is primarily produced as a by-product of bauxite processing in aluminum production and sphalerite refining in zinc extraction^3,4^. In the face of technological advancements, the current primary production has been challenged to meet market demands. Moreover, China’s dominance over the primary ore reserves up to 85%, raises significant concerns about supply stability, particularly in light of potential disruptions caused by pandemics, geopolitical tensions, trade embargoes, and the escalating influx of global e-waste^5^.

A significant portion of gallium in e-waste originates from light-emitting diodes (LEDs) used in electronic and lighting products^5^. LED chips are grown epitaxially mostly on sapphire substrates and rely on III/V semiconductors such as (Al,Ga,In)P, Ga(As,P), (In,Ga)N, and (Al,Ga)N, while REE-activated luminescent materials are often deposited onto the chips for the sake of LED chip spectra optimisation. Therefore, gallium, indium, and rare earth elements (REEs) are abundant in such LEDs^2^. The gallium content varies depending on the LED chip size and product type. For example, Surface-mounted device LEDs (SMD-LEDs) used in warm white LEDs exhibit higher concentrations of gallium, indium, silver, and gold compared to Ga(As,P)-based red LEDs^6^. However, due to the complex design of LEDs and other electronic devices, gallium is often lost during conventional e-waste recycling^7^. Studies have explored recycling gallium from secondary resources to address these challenges, particularly from the waste LED matrices. Nagy et al.(2017) optimized the recovery of Ga from white SMD-LEDs using mechanical shredding, ball milling, electrostatic separation, and chemical leaching with concentrated HCl, achieving up to 99% gallium enrichment in the non-conductive fraction^8^. Similarly, Swain et al.(2015) recovered 73.68% gallium fraction from the LED industry dust using comparable methods^9^. However, these studies did not provide detailed insights into the leaching mechanisms of gallium from waste precursors. As Zheng et al.(2024) noted, concentrated acids such as HCl dissolve not only critical metals like gallium, indium, and REEs but also base metals such as iron, copper, lead, and aluminum, complicating downstream purification steps^10^.

To overcome this lack of selectivity, organic acids have emerged as a promising alternative. According to the Hard-Soft Acid-Base (HSAB) theory, Ga (III):[Ar] 3d^10^, acts as a hard Lewis acid, showing a strong affinity towards hard bases such as oxygen donors in carboxylic acids^11^. This interaction is facilitated by available sites in Ga(III)’s 4 s, 4p, and 4 d orbitals, which allow for stable octahedral complexation with anionic ligands^12^. This thermodynamically robust binding was demonstrated by Zhou et al. (2019), in which organic acids such as oxalic acid and maleic acid could recover up to 95% of gallium from complex e-waste precursors^13^.

Furthermore, organic acids emerge as intriguing alternatives aligning with sustainable biotechnology, as many of these organic acids such as citric acid, succinic acid, lactic acid are readily produced by microbial cell factories on larger scales^14^. Owing to their versatility in their application as microbial cell factories, filamentous fungi species such as *Aspergillus niger* and *Penicillium sp.* have been the center of attention^15,16^. For instance, Parsa et al. (2024) optimized the critical metal recycling from the shredded LCD waste by implementing the supernatant of *Aspergillus niger*, which consisted of 14.9 g/L of oxalic acid, 1.2 g/L gluconic acid, 0.9 g/L citric acid, and 0.39 g/L malic acid^17^. Due to the superior efficiency of microbial metabolites in extracting metals from the e-waste, bioleaching has emerged as a sustainable and cost-effective alternative to traditional extraction methods^18^.

In this study, we investigated an alternative approach to gallium recycling using organic acids, focusing on gallium oxide as the precursor material. Our findings demonstrate that oxalic acid can leach gallium with comparable efficacy to concentrated HCl (4N), without any involvement of reducing agents. We elucidated the dissolution behavior of gallium, identifying the temperature as the most significant parameter, while underscoring its influence in pair-wise interaction involving acid concentration, and solid loading. Accordingly, we pinpointed the most optimized conditions for Ga recovery using a desirability study, achieving 37% of throughput recovery per processing step. The study sheds light on the gallium leaching kinetics and mechanisms, offering a novel perspective by modeling the concentration gradient during dissolution through regression analysis. Therefore, this research establishes a foundation for sustainable gallium recovery using organic acids, paving the way for bioleaching technology in the context of critical metal recycling. Our work aims to provide an outlook for the facilitation of subsequent purification steps and the enhancement of the efficiency of secondary recycling routes for e-waste materials.

## Methods

### Materials

All experiments were performed with Gallium (III) oxide ($\ge$99.99 purity) as the prime raw material purchased from Carl Roth GmbH + Co.KG. The leaching experiments for the screening test included the organic acids of oxalic acid, citric acid, malonic acid, maleic acid, malic acid, itaconic acid, glycolic acid, gluconic acid, and tartaric acid. In addition, reference experiments to the screening test were conducted using hydrochloric acid (HCl).

For the dissolved Ga amount quantification via UV-Vis spectroscopy, hexamethylenetetramine, cetyltrimethylammonium bromide, and pyrogallol red sodium salt were utilized. Calibration was performed using a Ga ICP standard solution (1000 mg/L, Carl Roth GmbH + Co.KG at 1000 mg/L). To maintain consistency, the pyrogallol red reagent was replenished every two months.

All reagents were of analytical grade and used without further purification.

### Leaching experiments

Leaching experiments were conducted in 500 mL three-necked round-bottom flasks equipped with a reflux condenser. The temperature during leaching was held constant ($\pm 2^\circ$C) using a *Heidolph Mix’n’Heat Core* hot plate, equipped with an integrated heating block and temperature sensor. To prevent contamination from extraneous ions and ensure precise thermal control, the temperature sensors were encased in glass sleeves. All leaching solutions were stirred at 450 rpm using magnetic stirrers.

The interval sampling of 2 ml for Ga(III) concentration was executed after 15 min, 30 min, and 1, 2, and 3 hours. The collected samples were centrifuged for 15 minutes at 14,000 rpm to separate the captured solid samples from the leached liquor. The samples were diluted and stored in the cooling chamber at $4^\circ$C until the UV analysis.

#### Screening test

In the screening tests, the aforementioned organic acid leaching agents were compared on their ability to extract gallium (Ga) from the precursor material (mg/L). Prior to the experiments, each organic acid solution was prepared at a concentration of 0.01 M. To decouple the influence of proton activity through dissociation from ligand complexation, another set of experiments with the same reagents, organic acid, were adjusted to pH 1.2 using HNO_3_ instead of HCl to prevent interference through inadvertent speciation.

In addition, control experiments using 0.063 M HCl (pH 1.2) were included to establish a baseline for proton-driven leaching (acidolysis) in the absence of organic ligands. This reference enabled the differentiation between purely acid-induced dissolution and ligand-assisted complexolysis in the organic acid systems.

For all screening experiments, the solid loading was set to 10 g/L. Accordingly, gallium oxide (1.00 ± 0.02 g) was added to 100 mL of the pre-heated leaching solution as soon as the reaction temperature reached $90^\circ$C. The leaching was carried out under these conditions for 3 hours before terminating the experiment.

#### OFAT experiments

Building upon the screening test, the one-factor-at-a-time (OFAT) experiments were performed using the most effective leaching agent identified through the screening test. In these experiments, the influence of pulp density, temperature, acid concentration, and reaction time was studied individually. Throughout all OFAT tests, the solution volume was maintained at 100 ml, and agitation was kept constant at 450 rpm.

For each test, only the parameter under investigation was varied, while the others were held constant at their standard values:

- Temperature: Varied between 45 and $90^\circ$C, with 1 M acid concentration, 10 g/L solid loading, and 3 hours of reaction time. The range was determined to be in this range, as below $45^\circ$C, a significant Ga leaching could not be observed, nor above $90^\circ$C due to the excessive evaporation.
- Acid concentration: Varied from 10 to 1000 mM, under conditions of $90^\circ$C, 10 g/L solid loading, and 3 hours of reaction time.
- Solid loading varied between 5 and 20 g/L at $90^\circ$C and 1 M acid concentration for 3 hours.
- Reaction time: The time-dependent experiments were conducted through interval sampling at predetermined intervals up to 3 hours. No further increase in leaching yield was observed beyond this point.

#### Characterization and analysis

The dissolved Ga amount was determined using UV spectrophotometry Evolution 201 UV-VIS from Fischer Scientific, based on the protocol developed by Wyganowski (1981)^19^. All measurements were performed in quintuplicate to ensure statistical reliability. Deionized Water was used in the blank measurements.

In addition to the measurement of Ga(III) concentration, the recovery rate of Ga (%L) was calculated using the following equation, adapted from Cai et al. (2019)^20^:

1 $$\begin{aligned} L\% = C_{M_{leaching solution}}/C_{M_{HCl}}x100 \end{aligned}$$

$C_{\mathrm {M,\,leaching solution}}$ is the concentration of dissolved Ga in the leaching solution from the precursor material, whereas $C_{\mathrm {M\,HCl}}$ denotes the reference concentration of dissolved Ga in the solution using concentrated HCl (4 M).

The pH of the leaching solutions was measured before and after each experiment using a calibrated Dostmann pH meter. The particle size of the precursor material was analyzed in triplicate via wet dispersion using the Microtrac S3500 Particle Size Analyzer.

### Kinetic studies

Leaching kinetics were modeled based on the shrinking core model (SCM) theory, where the leaching recoveries calculated at varying temperatures were fitted to models representing chemical reaction control, diffusion through gas-film control, and ash layer control models over time (equations (1), (2), and (3) were outlined in the *Supplementary Material*). Based on the deduced optimal conditions from the OFAT results yielding the highest extraction for Ga, the experimental conditions for these kinetic studies involved varying only the temperature, while the other parameters were held constant at an oxalic acid concentration of 1000 mM, a pulp density of 10 g/L, and a leaching duration of 3 hours.

The regression analysis was done using Python 3 via the *LinearRegression* function imported from the sci-kit-learn library. The SCM correlations were linearized via implementation of parameters and variable (leaching efficiency (%) as denoted by x). The regression coefficient and the rate constants were determined accordingly for each linearized expression characterizing reaction-limited, diffusion-limited and ash layer-limited SCM correlations corresponding to the leaching duration (time (h)) at temperature levels ranging from $45-90^\circ$C. Subsequently, the activation energy was inferred from the slope of the natural logarithm of the rate constant vs. 1000/T.

### Design of experiments and process optimization

A response Surface Methodology (RSM) based on a Box–Behnken design was employed to assess the interactions between key process variables and improve Ga recovery. The experimental matrix included 27 runs with three replicates at the center point. The BBD approach was chosen due to the limited number of experimental parameters and the resulting small dataset.Since BBD provides robust estimation of linear, interaction, and quadratic effects with a minimal number of experiments, making it an efficient and statistically rigorous choice for optimizing Ga leaching under the experimental constraints of this study. The studied variables in the BBD were acid concentration, pulp density, temperature, and leaching time.

The response data were modeled using a reduced quadratic equation, and the significance of model terms was evaluated using ANOVA with a confidence level of 95% (p<0.05). Statistical modeling and analysis, including the calculation of regression coefficients, evaluation of model adequacy, and generation of surface plots were conducted in Python 3 and validated using Minitab 17 software. The model fitting was performed via ordinary least squares regression with a prior normalization of variables, with the quadratic model yielding the best description of the system behavior (see Supplementary Table S8). Although the initial ANOVA suggested significant effects, the lack-of-fit test indicated unacceptable deviations. Consequently, six additional center point runs were included, which significantly improved model fit. The refined quadratic model was then used for subsequent analysis.

Based on the final ANOVA results, significant main effects and two-way interactions (p<0.05) were visualized using 2D response surface plots under standard conditions. For the visualization of the pair-wise interaction plots, the hold values for the non-visualized parameters were held constant at their median values designated the BBD scaling and to reflect the Ga extraction (g/L) in the same scaling, the adjustment was implemented for a fair comparison. Furthermore, the optimal recovery conditions for Ga leaching was pinpointed by the desirability-based scenario optimization in Minitab 17 and verified via the demo version using Stat-Ease Sofware. Multiple scenarios were explored to balance leaching efficiency with practical constraints for scale-up. After defining optimal criteria, confirmation experiments designated by point-prediction analysis were carried out in triplicate. The experimental results fell within the 95% confidence interval, verifying the accuracy of the model predictions.

## Results

### Screening test

In the study, a diverse range of organic acids (refer to S1) were evaluated in terms of their performance to extract gallium from insoluble materials. What is more, to delineate the effect of proton attack (acidolysis) and complexation (complexolysis) in the leaching process, the experiments were performed under neutral pH and standardized pH of 1.2, as this value was determined to be the lowest possible value applicable to observe the neutral state across a variety of the screened acids in the study.

**Fig. 1:**
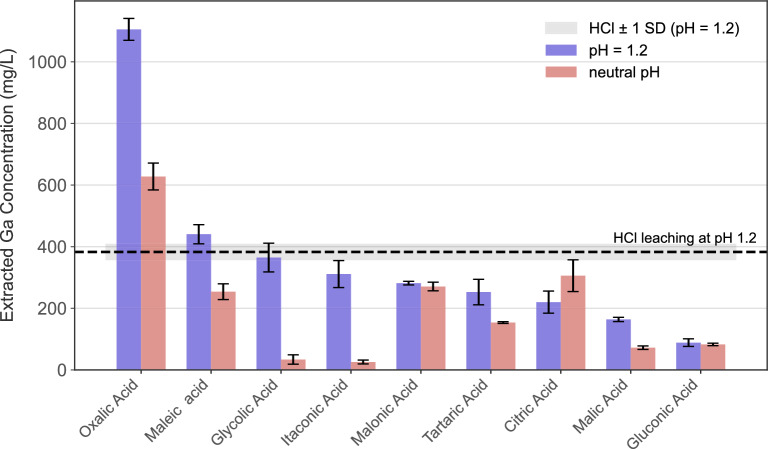
Comparison of gallium (Ga) extraction by various organic acids under two different pH conditions. The bar chart shows the Extracted Ga Concentration (mg/L) for each acid at its neutral pH and at a standardized pH of 1.2. All experiments were conducted under fixed conditions: an acid concentration of 10 mM, a solid-to-liquid (S/L) ratio of 10 g/L, a temperature of $90^\circ$C, and a duration of 3 hours. The dashed line represents the baseline (control) leaching performance of hydrochloric acid (HCl) at pH 1.2, with the shaded grey area indicating ±1 standard deviation (SD).

As shown in Fig. 1, oxalic acid reveals the highest efficacy in Ga extraction, achieving a concentration of 1105.2 ± 61.4 mg/L, followed by maleic acid at 440.3 ± 53.9 mg/L. It is apparent from Fig. 1 that, oxalic acid outperformed all the other tested acids by a factor ranging from approximately 2.5 to 10. As the leaching efficiency of maleic acid and oxalic acid surpassed the baseline experiments from purely proton-attack based HCl leaching, the findings suggest a synergistic effect of these acids comprising acid attack and complexation effect in Ga leaching.

In Fig. 1, a significant improvement was observed after adjusting the pH to 1.2 for glycolic and itaconic acid, indicating acidolysis through external proton donation, while complexolysis deemed to be negligible at the neutral pH. On the other hand, malonic acid, tartaric acid, and citric acid formed complexes at the neutral pH (complexolysis), which can be linked to higher dissociation at the neutral pH (see S1). As the pH was adjusted to 1.2, the extraction is directly overruled by the acidolysis through proton attack. Surprisingly, the pH adjustment to 1.2 diminished the Ga extraction for citric acid, which can be attributed to the full protonation of acid and consequent buffering effect. Moreover, weaker leaching agents of malic and gluconic acids did not perform substantial Ga extraction ($<200 mg.L^{-1}$) under both conditions, rendering them poor Ga leachants.

All in all, the screening test revealed the performance of the organic acids under varying pH conditions. In general, monodentate acids such as glycolic and itaconic acids depended solely on the proton attack to extract Ga, whereas multidentate ligands such as citric, malonic and tartaric acids exhibited moderate leaching performance through the complexation of gallium metal ions and deprotonated ligands at the neutral pH. Conversely, oxalic acid and malic acid benefited from the enhanced leaching through the synergistic influence of acid attack and complexolysis through chelation effect. Among all the screened acids, oxalic acid attained the highest extraction up to 10 times the weakest acid and roughly three times the extraction attained by the inorganic leaching HCl (pH=1.2), pinpointing it as the most effective leaching agent for Ga extraction. Therefore, oxalic acid was selected as the primary organic reagent for subsequent experiments in this study.

### One-factor-at-a-time (OFAT) experiments

**Fig. 2:**
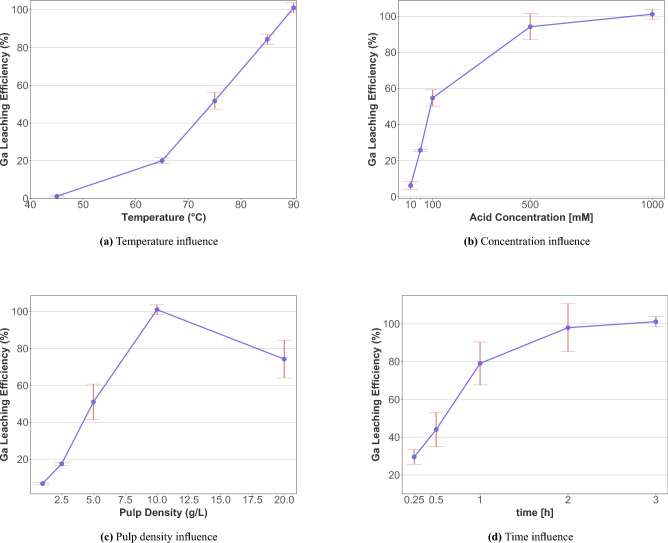
One-factor-at-a-time (OFAT) analysis of gallium (Ga) leaching efficiency with oxalic acid. The plots show the Ga leaching Efficiency (%) in response to the tested parameters in OFAT: (**a**) Temperature (°C), (**b**) Acid Concentration (mM), (**c**) Pulp Density (g/L), and (**d**) time (h). For each plot, the parameter on the x-axis was varied while the other three were held constant at standard conditions: T= $90^\circ$C, [A] = 1000 mM of oxalic acid, S/L= 10 g/L pulp density, and t=3 hours.

Following the identification of the optimal leaching agent for Ga, influence of key experimental parameters including reaction temperature, acid concentration, pulp density, and reaction duration was systematically evaluated using one-factor-at-a-time (OFAT) experiments as detailed in Section: *Methods*. The responses of each parameter on Ga extraction efficiency are presented in Fig. 2.

Oxalic acid leaching at varying temperatures as shown in Fig. 2a, indicated negligible Ga dissolution. As the temperature is elevated from $60^\circ$C onwards, Ga extraction increased gradually, culminating in the full conversion at $90^\circ$C. These results indicate the Ga extraction can take full effect once the activation energy is surpassed and reach a maximum at $90^\circ$C.

In another set of experiments dealing with acid concentration as given in Fig. 2b, Ga leaching was enhanced as the higher acid concentrations were incorporated, indicating a contribution through the abundance of active sites, stimulating a deprotonation and complex formation. The Ga extraction reached a plateau between 500 and 1000 mM, as all the desired Ga amount was leached into the solution.

In assessing the influence of pulp density at 5, 10, and 20 g/L. As shown in Fig. 2c, the solid dumping at 10 g/L indicated a maximum conversion. Yet, increasing loadings reaching 20 g/L led to the drop in the Ga extraction by 25%. These findings are deemed to be associated with the excessive acid consumption through pulp overloading, impeding the metal extraction due to higher viscosity, as well. Following this, in order to determine the leaching duration for full conversion, the leaching reaction was observed for a certain amount of time through interval sampling. According to the data in the Fig. 2d, the oxalic acid leaching at standard conditions exhibited a max conversion in 3 hours.

### Kinetics of gallium leaching and optimization

**Fig. 3:**
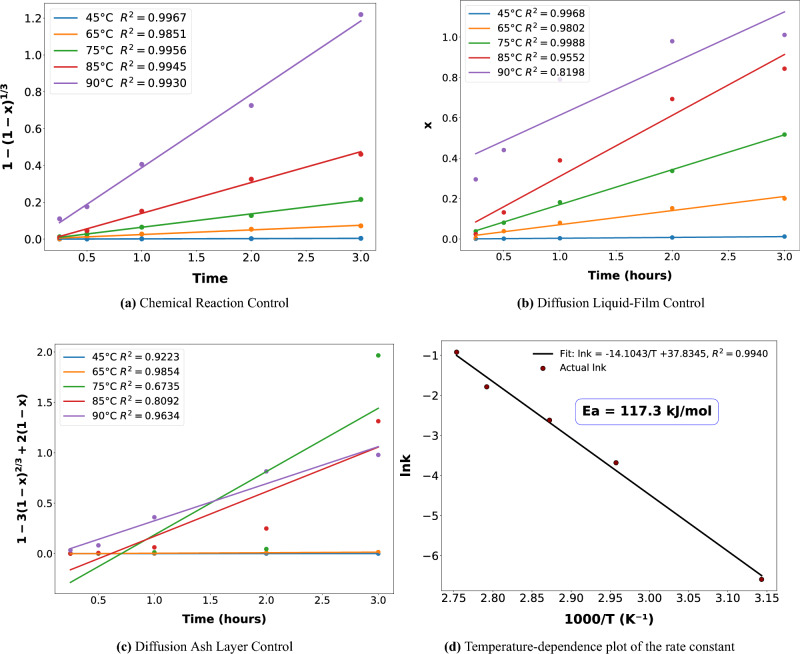
Ga conversion Kinetic Models based on SCM correlations and parameters (outlined in detail in the *Supplementary Material*) at different temperature ranges [$45-90^\circ$C]. Hold-values for the remaining parameters inferred in accordance with the findings of the OFAT experiments (acid concentration=1000 mM, S/L = 10 g/L, time=3 hours).

To investigate the Ga dissolution kinetics by oxalic acid, Levenspiel’s shrinking core model (SCM) was chosen, as it is widely recognized for depicting fluid-solid interactions^21^. Accordingly, Ga conversion data at varying temperatures of 45, 60,75, and $90^\circ$C were fitted to SCM correlations (refer to the Supplementary material equations (1),(2), and (3) and for material specifications Supplementary Table S3). The comprehensive model fitting data are outlined in the Supplementary Table S2.

According to the Fig.3a and the fitting summary in the Supplementary table S2, chemical reaction control achieved almost a perfect fit ($R^{2}\ge$0.99) at each designated temperature. Concerning the film-diffusion control, as shown in Fig. 3b, the model could capture the dissolution behaviour of Ga between 45 and $75^\circ$C, implying a mass transfer through film diffusion at lower temperatures. On the other hand, the data in Fig. 3c could not correlate to the ash-layer diffusion control model, suggesting negligible intermediate product built-up.

Arrhenius plot in Fig. 3d indicated a relatively higher activation energy at 117.3 kJ.mol^−1^, suggesting the presence of a significant energy barrier that can only be overcome at elevated temperatures, aligning with the temperature-sensitive chemical control as a limiting step. Accordingly, the data in the Supplementary Table S2 indicated a higher residence time for full conversion at $45 ^\circ$C at $\tau$ = 244.38 h. As the temperature is elevated from $75^\circ$C towards $90^\circ$C, the chemical reaction controls the dissolution, overcoming the threshold energy barrier, which reduces the time for total conversion to 2.51 h. So the findings suggest a diffusion-controlled mass transfer at lower temperatures, which is limited at higher temperature through the surface chemical reaction control. This outcome corroborates the OFAT results, confirming temperature as a critical limiting factor that can be overcome by operating at elevated levels.

### Optimization

For evaluating the parameter interactions beyond the scope of OFAT, Box-Behnken Design (BBD) was implemented to elucidate the gallium dissolution through response-surface modelling. Accordingly, BBD yielded 27 experiments as well as including 3 replicates around central points to asses the influence of the parameters stated in the Supplementary Table S4 and the Supplementary Table S5, respectively. The order of experiments as given in the Supplementary Table S4 was randomized twice to enhance the external validity of the experimental design. The designated experimental points are outlined in the Supplementary Table S7.

**Table 1:** ANOVA summary for the quadratic response surface model of Ga leaching.

Adjusted R-squared	0.838
F-statistics	12.78
p-value	1.33E-06
No. Of Observations	33
DF residuals	18
DF model	14
Adequate Precision (S/N)	13.49
Lack of Fit (p-value)	0.517

According to the model-fit results outlined in the Supplementary Table S8, Ga dissolution exhibited non-linear characteristics, as the data aligned with the quadratic model the best. Despite showing significance, BBD needed to be refined, as the previous design yielded a significant level of Lack-of-Fit due to the invariance in the outcomes of the designated center points. After addressing this “Over-fitting” conundrum through increasing the number of center points to 6 (refer to the Supplementary Table S6), the output data provided in Table 1 showed accordance with the quadratic model at p = 1.33e-06 < 0.05.

The predicted Ga extraction based on the quadratic model yielded the mathematical expression characterizing the Ga extraction (g/L): Extracted Ga (g/L) = 4.8647 + 0.0047(**A**) + 0.1519(**B**) + 0.2356(**C**) + 0.9199(**D**) + 0.0033(**C**^**2**^) + 0.0003(**A** $\times$ **C**) + 0.0071(**B** $\times$ **C**), where A, B, C, and D correspond to the respective process parameters listed in the Table S5.

**Fig. 4:**
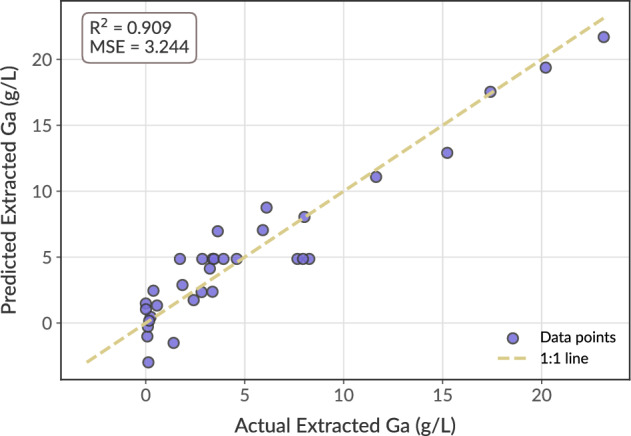
Parity plot of the quadratic model fitted into BBD optimization for Ga extraction, demonstrating a relation between the predicted values by the model and the experimental data, as the model fitting indicated by the regression coefficient R^2^=0.909.

The precision plot given in Fig. 4, confirmed the accuracy of the quadratic model through model fitting. The results in the Table 1 also indicated a curvature stemming from the significance of the term C: Temperature, whilst the remaining parameters did not indicate any significance in the quadratic terms

**Fig. 5:**
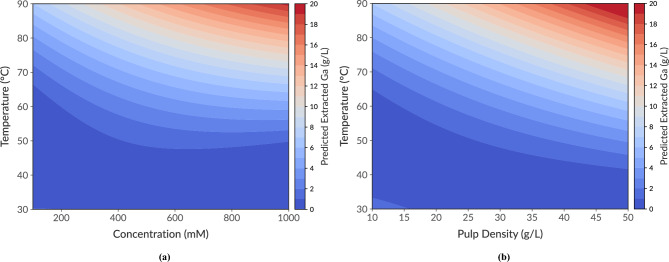
Response surface contour plots illustrating the significant interaction effects on predicted gallium (Ga) extraction (g/L). The plots show the combined effects of (**a**) temperature and acid concentration, and (**b**) temperature and pulp density. For each plot, variables not shown were held constant at their central values: pulp density (30 g/L) and time (5 h) for (**a**); acid concentration (550 mM) and time (5 h) for (**b**).

In support of these findings, Fig. 5 provides 2D response surface plots illustrating only the statistically significant interactions from the Box-Behnken design (BBD), specifically Temperature $\times$ Concentration and Temperature $\times$ Pulp Density. To effectively demonstrate each interaction, the other parameters were maintained at their central values. As indicated in the plots, gallium extraction efficiency significantly improved at elevated temperatures due to synergistic interactions. However, despite increases in either concentration or pulp density, gallium extraction showed minimal improvement unless the reaction temperature exceeded approximately $50^\circ$C.

Subsequently, the desirability analysis was conducted to determine the best possible outcome in the Ga extraction. As a result, the simulation evaluated 4,096 different parameter combinations among which the scenarios were ranked for the selection criteria favoring lower concentration, higher pulp density, lower temperature, and lower processing time for the sake of higher Ga extraction. The desirability index of 0.457 was found to be the best possible outcome for the experimental conditions yielded 710. 55 mM of oxalic acid concentration, operation temperature at $84.5^\circ$C, 50 g/L loading, and 3.2 hours(see Supplementary Figure S3).

Accordingly, the optimization based on the desirability analysis predicted a gallium concentration of 18.5 g/L under optimal conditions, which was subsequently validated experimentally through replicate trials. The experimental results confirmed this prediction, with the measured gallium concentration falling within the 95% confidence interval of the predicted value.

In our study, the leaching process is prolonged to 3.2 hours, owing to having a larger throughput at 50 g/L. Under optimized conditions, 37% of the loading can be leached per operation. Even though a substantial amount of loading can be converted into soluble products, the remaining residue can be post-processed in regenerated oxalic acid after the recovery of Ga as pure metal.

## Discussion

This study systematically investigated the Ga dissolution, with the screening test revealing the superior performance of oxalic acid in the leaching process. Our findings attribute the efficacy of oxalic acid to its dual functionality in the Ga extraction, comprising acidolysis through proton attack solubilizing the precursor, as well as forming stable complexes through complexolysis step.

The screening test comparing Ga leaching at neutral pH versus a standardized pH of 1.2, successfully distinguished between different leaching mechanisms delineating acidolysis, complexolysis, and, in some cases, a synergistic dual effect across a wide spectrum of organic acids. Results in Fig. 1 suggested that monodentate candidates ligands such as glycolic acid, could only extract Ga at the lower pH through external protons supplied via pH adjustments, indicating a mechanism relying almost exclusively on acidolysis driven by external protons with negligible complexation. Thereby, monodentate ligands were outperformed in terms of Ga extraction by multidentate ligands. Similarly, itaconic acid, despite being a bidentate ligand, exhibited poor steric binding for chelate formation for successful complexation, thus failing to satisfy the requirements for stable complex formation according to HSAB theory.

Conversely, multidentate ligands such as citric, malonic and tartaric acids performed better at their neutral pH, demonstrating leaching primarily through complexolysis. Concerning the Ga extraction by these ligands, the addition of external protons for pH adjustment impeded complex formation by protonating the active ligand sites. In contrast, oxalic acid and maleic acid demonstrated a synergistic interaction of acidolysis and complexolysis during Ga leaching. These acids not only form stable complexes at neutral pH but also enhance leaching efficacy through pH adjustment.

Specifically, oxalic acid achieved the highest and distinctly most selective extraction of Ga. The dual functionality of oxalic acid as both a strong acid and an excellent Lewis base makes it a superior alternative to harsh conventional inorganic acids such as HCl ($pK_a = -6.0$) and H_2_SO_4_ ($pK_a1 = -3.0$). Furthermore, its ability to selectively precipitate other metals, including rare earth elements (REE), Fe(II), and Cu(II) and so on, underscores its potential as a strategic and versatile for complex waste processing^13,22^. This makes oxalic acid a suitable candidate for developing more selective and environmentally benign recovery technologies.

Having identified oxalic acid as the optimal leaching agent from the screening test, the dissolution kinetics were characterized utilizing the shrinking core model (SCM). The findings concerning the dissolution kinetics of Ga, based on data from the One-Factor-at-a-Time (OFAT) experiments, pointed out that Ga dissolution is driven by a synergistic interplay of film diffusion and surface chemical reaction. At lower temperatures ($45-75^\circ$C), film diffusion governed the mass transfer of Ga from the solid phase into the liquid medium, whereas at elevated temperatures ($75-90^\circ$C), the chemical surface reaction controlled the dissolution kinetics. These findings align with the argument presented by Levenspiel (1998), who emphasized that dissolution phenomenon should not be attributed solely to a single rate-limiting step but rather to the combined influence of two or more controlling stages^21^.

Extending the scope of leaching beyond the comparison of different agents and the temperature dependence analyzed in the SCM, the OFAT experiments proved to be of paramount importance. In particular, the temperature variations reiterated the correlations observed in the kinetic study and highlighted the necessity of elevated temperatures to overcome the energy barrier at 117.3 kJ/mol. As particle collision frequency is commensurate with increasing temperature, Ga recovery could be maximized at $90^\circ$C. In addition, the OFAT on concentration influence revealed a saturation effect, as available active sites were progressively consumed starting from an initial acid concentration at 500 mM.

From a techno-economic standpoint, utilizing higher solid loadings in leaching operations is advantageous, as higher throughput per leaching step can reduce overall material and processing costs. Nonetheless, the OFAT findings indicated that higher loadings reaching 20 g/L of pulp density impeded Ga extraction, attributed to increased viscosity and the precipitation of oxalate complexes. At the throughput of 10 g/L, the active sites of the acid appeared to be fully utilized, whereas further increases beyond 10 g/L led to acid overconsumption and mass transfer limitations, consistent with the observations of Zhou et al.(2019)^13^. Thus, the findings of OFAT highlighted a trade-off, in which higher pulp densities are desirable for one-pot operations, on the other hand having higher solid loadings per operation poses challenges in the extraction when the acid concentration and temperature and a suitable exposure time are not adjusted accordingly.

To address the limitations observed in the OFAT experiments and to empirically characterize Ga dissolution under optimized conditions, a Box-Behnken Design (BBD) was adopted. This approach encapsulated parameter interactions while aligning with techno-economic considerations, aiming for maximizing throughput while ensuring sufficient acid concentration, optimal temperature as well as process duration. The BBD analysis identified a curvature driven by temperature and synergistic interactions between temperature x pulp density and temperature x acid concentration, yielding a significant model (p $< 0$.05).

Based on these insights from BBD analysis, a desirability index was applied to identify conditions that maximize Ga extraction per operation while minimizing energy and reagent consumption. Thereby, this methodology mitigates the shortcomings observed in the results of OFAT, where higher pulp densities undermined Ga extraction due to mass-transfer limitations and overconsumption of active acid sites. By incorporating parameter interactions, the BBD enabled a robust approach on a constrained dataset to pinpoint suitable condition, that can be tailored for large-scale operations. Under these coupled conditions, a one-pot strategy with 50 g/L solid loading could maximize Ga recovery while reducing the overall energy and reagent demand per unit of processed material. A subsequent point-prediction analysis based on this desired scenario at 710.55 mM acid concentration, $84.5^\circ$C, 50 g/L of solid loading within 3.2 hours, yielded a maximum conversion of 37% per operation.

Overall, this study provides a comprehensive examination of Ga dissolution, extending the discussion beyond conventional methodologies. By systematically evaluating potential leaching candidates and elucidating the dissolution mechanism, this study underscores the singular influence of core parameters whilst indicating the multifaceted optimization strategy including parameter interactions to reconcile aforementioned limitations. The implementation of pure substances enabled the exclusion of interference of competing ions, thereby adopting a more targeted and enhanced extraction in juxtaposition to previous studies, as well as notably surpassing the results obtained by Zheng et al. (2024), who achieved a dissolution of 150 µmol/L Ga from identical precursor materials using the siderophore reagent desferrioxamine E in 25 days^10^.

As this study focused on Ga alone for the sake of providing more clarity and mechanistic insights, future research should explore the sequential effects of co-existing metal ions commonly found in ore and electronic waste matrices to develop selective recovery strategies using oxalic acid, not only for gallium but also for other critical elements such as indium and iron(III), potentially interfering with Ga dissolution. Given that oxalic acid can be biogenically produced by microorganisms such as *Aspergillus Niger*, the integration of bioprocess technologies with metal leaching processes offers a promising and sustainable approach. Furthermore, the incorporation of post-processing applications, including acid regeneration and pure metal recovery through adsorption, precipitation, and solvent extraction, is essential for establishing closed-loop systems for the efficient recovery of critical metals from various precursors.

## Supplementary Information

Supplementary Information.

## Data Availability

All data generated or analyzed during this study are provided in the published article and the accompanying supplementary material.

## References

[1] Baldé, C. P. *et al.* The global e-waste monitor. *United Nations University (UNU), International Telecommunication Union (ITU) & International Solid Waste Association (ISWA), Bonn/Geneva/Vienna* 1–109 (2017).

[2] De Oliveira, R., Benvenuti, J. & Espinosa, D. A review of the current progress in recycling technologies for gallium and rare earth elements from light-emitting diodes. *Renew. Sustain. Energy Rev.* **145**, 111090 (2021).

[3] Lu, F. et al. Resources and extraction of gallium: A review. *Hydrometallurgy* **174**, 105–115 (2017).

[4] Greber, J. F. Gallium and gallium compounds. *Ullmann’s encyclopedia of industrial chemistry* (2000).

[5] Robart, M., Zhang, A. & Peek, E. Gallium production from primary and secondary sources. In *Conference of Metallurgists*, 1383–1397 (Springer, 2024).

[6] Vinhal, J. T., de Oliveira, R. P., Coleti, J. L. & Espinosa, D. C. R. Characterization of end-of-life leds: Mapping critical, valuable and hazardous elements in different devices. *Waste Manag.* **151**, 113–122 (2022). 35939950 10.1016/j.wasman.2022.07.027

[7] Ueberschaar, M., Otto, S. J. & Rotter, V. S. Challenges for critical raw material recovery from weee-the case study of gallium. *Waste Manag.* **60**, 534–545 (2017). 28089397 10.1016/j.wasman.2016.12.035

[8] Nagy, S., Bokányi, L., Gombkötő, I. & Magyar, T. Recycling of gallium from end-of-life light emitting diodes. *Arch. Metall. Mater.* **62**, 1161–1166 (2017).

[9] Swain, B. et al. Recycling process for recovery of gallium from gan an e-waste of led industry through ball milling, annealing and leaching. *Environ. research* **138**, 401–408 (2015). 10.1016/j.envres.2015.02.027 25769129

[10] Zheng, K., Benedetti, M. F., Jain, R., Pollmann, K. & van Hullebusch, E. D. Recovery of gallium (and indium) from spent leds: Strong acids leaching versus selective leaching by siderophore desferrioxamine e. *Sep. Purif. Technol.* **338**, 126566 (2024).

[11] Weber, B. & Birgit, W. Coordination chemistry. *Basics Curr. Trends.* (Springer, Berlin, Germany, 2023).

[12] Wood, S. A. & Samson, I. M. The aqueous geochemistry of gallium, germanium, indium and scandium. *Ore geology reviews* **28**, 57–102 (2006).

[13] Zhou, J. et al. Recovery of gallium from waste light emitting diodes by oxalic acidic leaching. *Resour. Conserv. Recycl.* **146**, 366–372 (2019).

[14] Di Lorenzo, R. D., Serra, I., Porro, D. & Branduardi, P. State of the art on the microbial production of industrially relevant organic acids. *Catalysts* **12**, 234 (2022).

[15] Esmaeili, A. et al. Simultaneous leaching of cu, al, and ni from computer printed circuit boards using penicillium simplicissimum. *Resour. Conserv. Recycl.* **177**, 105976 (2022).

[16] Bahaloo-Horeh, N. & Mousavi, S. M. Analyzing the effects of culture media additives on oxalic acid bioproduction for use in metal bioleaching. *Waste and Biomass Valorization* **15**, 2687–2703 (2024).

[17] Parsa, A., Horeh, N. B. & Mousavi, S. M. A hybrid thermal-biological recycling route for efficient extraction of metals and metalloids from end-of-life liquid crystal displays (lcds). *Chemosphere* **352**, 141408 (2024). 38336041 10.1016/j.chemosphere.2024.141408

[18] Brandl, H., Bosshard, R. & Wegmann, M. Computer-munching microbes: metal leaching from electronic scrap by bacteria and fungi. *Hydrometallurgy* **59**, 319–326 (2001).

[19] Wyganowski, C. Spectrophotometric determination of aluminium and gallium with pyrogallol red and cetyltrimethylammonium ions. *Microchem. J.* **26**, 45–50 (1981).

[20] Cai, C., Fajar, A. T., Hanada, T., Wakabayashi, R. & Goto, M. Amino acid leaching of critical metals from spent lithium-ion batteries followed by selective recovery of cobalt using aqueous biphasic system. *ACS omega* **8**, 3198–3206 (2023). 36713728 10.1021/acsomega.2c06654 PMC9878538

[21] Levenspiel, O. *Chemical reaction engineering* (John wiley & sons, 1998).

[22] Schmitz, D., Prasetyo, H., Birich, A., Yeetsorn, R. & Friedrich, B. Co-precipitation of metal oxalates from organic leach solution derived from spent lithium-ion batteries (libs). *Metals* **14**, 80 (2024).

