# Supplementary material for: Fundamental insights into gallium leaching for sustainable electronic waste recovery
Source: Sci Rep. 2025 Dec 2;15:43023. doi: 10.1038/s41598-025-30908-3 (PMC12675773; doi:10.1038/s41598-025-30908-3)
Supplement: Supplementary file 1 — Supplementary Information. [file 41598_2025_30908_MOESM1_ESM.pdf]

Supplementary Material

Organic acid	Molecular formula	pK_{a1}	Theoretical pH	Initial pH	f_H (pH = initial pH)	f_H (pH = 1.2)
Oxalic acid	$C_2H_2O_4$	1.25	2.59	2.28	0.92	0.47
Citric acid	$C_6H_8O_7$	3.13	2.62	2.41	0.16	0.012
Maleic acid	$C_4H_4O_4$	1.9	2.18	2.09	0.6	0.16
Malic acid	$C_4H_6O_5$	3.46	2.77	2.67	0.14	0.005
Gluconic acid	$C_6H_{12}O_7$	3.6	2.83	2.8	0.14	0.004
Malonic acid	$C_3H_4O_4$	2.83	2.5	2.35	0.25	0.023
Tartaric acid	$C_4H_6O_6$	3.03	2.58	2.51	0.23	0.015
Glycolic acid	$C_2H_4O_3$	3.83	2.94	2.76	0.078	0.002
Itaconic acid	$C_5H_6O_4$	3.63	2.85	2.77	0.12	0.004

Table S1. Overview of the screened organic acids, their chemical properties and protonation behaviour at pH = 1.2

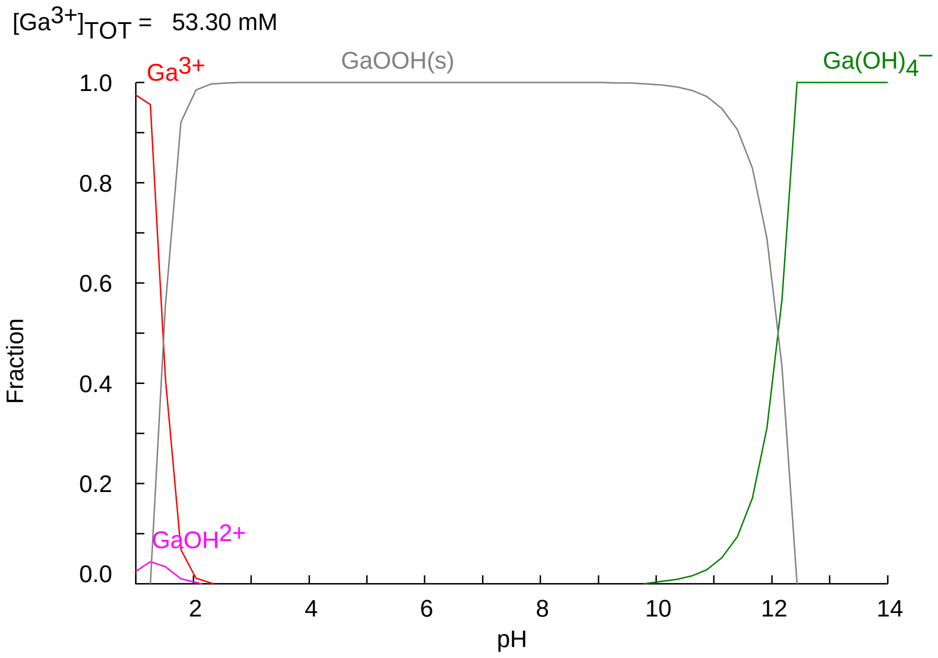

Figure S1. Gallium speciation at 10 g/L of solid loading over a wide pH range

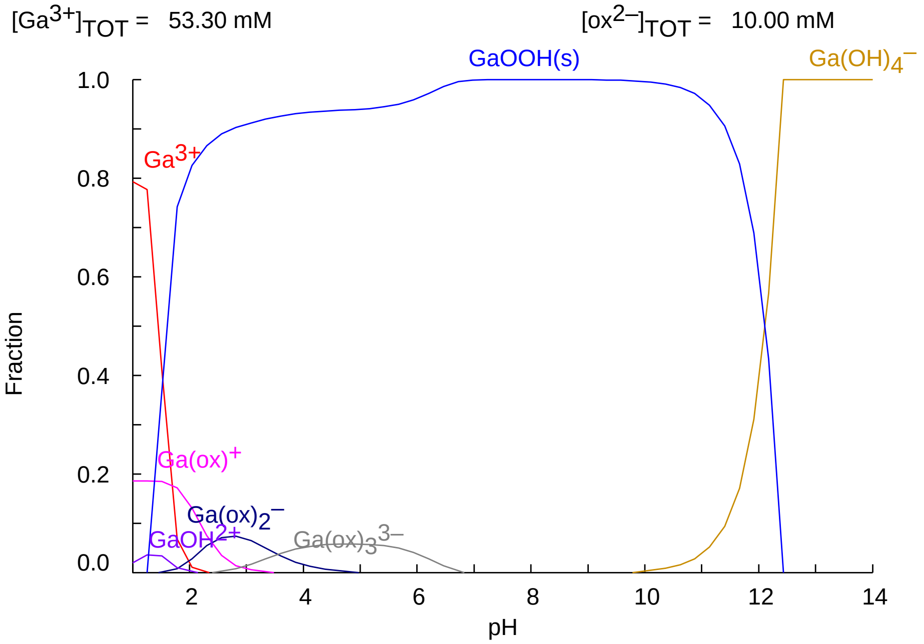

Figure S2. Gallium speciation in 10 mM oxalic acid solution at 10 g/L of solid loading over a wide pH range

Kinetic modelling based on Shrinking Core Model by Levenspiel

1)Reaction-limited: $$\frac{t}{\tau} = 1-(1-x_B)^{\frac{1}{3}}, \qquad \tau = \frac{\rho}{bk''C_{AL}} \tag{1}$$

2) Diffusion-limited: $$\frac{t}{\tau} = x_B, \qquad \tau = \frac{\rho R}{3bk_L C_{AL}} \tag{2}$$

3) Ash layer-limited: $$\frac{t}{\tau} = 1-3(1-x_B)^{\frac{2}{3}}+2(1-x_B), \qquad \tau = \frac{\rho R^2}{6bD_{eff}C_{AL}} \tag{3}$$

x_B : Fraction of B (solid reactant) reacted

t : Time [s]

τ : Elapsed time for complete conversion

ρ_b : Material density [mole/m^3]

b : Mole ratio of solid (B) to reactant(A)

C_{AL} : Concentration of A in bulk liquid [mol/m^3]

k'' : Surface reaction rate constant [m/s]

k_L : Liquid-film mass transfer coefficient [m/s]

D_{eff} : Effective diffusivity through ash/product layer [m^2/s]

R_0 : Initial particle radius [m]

Table S2. Kinetic parameters based on different SCM control models for Ga_2O_3 dissolution

**SCM Model**	**Chemical Reaction Control**				**Diffusion Control**				**Ash Layer Control**			
**Temp (°C)**	Equation	R^2	τ	k''	Equation	R^2	τ	k_L	Equation	R^2	τ	D_{eff}
45	$y = 0.0014x - 0.0002$	0.997	730	4E-06	$y = 0.0041x - 0.0004$	0.996	244.38	4.00E-06	$y = 0.000017x - 0.000009$	0.92	57609	0
65	$y = 0.0252x - 0.0005$	0.985	39.72	7E-05	$y = 0.0699x + 0.0008$	0.980	14.3	6.50E-05	$y = 0.0055x - 0.0022$	0.985	182.4	7.52E-05
75	$y = 0.0728x - 0.0086$	0.996	13.74	2.03E-04	$y = 0.1728x - 0.0024$	0.998	5.78	1.60E-04	$y = 0.6281x - 0.4428$	0.670	1.59	8.61E-03
85	$y = 0.1675x - 0.0278$	0.994	5.97	4.66E-04	$y = 0.3015x + 0.0091$	0.955	3.32	2.80E-04	$y = 0.4427x - 0.2715$	0.810	2.26	6.07E-03
90	$y = 0.3982x - 0.0102$	0.992	2.51	1.11E-03	$y = 0.2554x + 0.3583$	0.820	3.91	2.37E-04	$y = 0.3677x - 0.0423$	0.960	2.72	5.04E-03

Table S3. Specifications of gallium oxide particles

g_b	31.37
R_0	2.96e-05
b	0.333
C_{A_L}	1000

Table S4. Overview of Box-Behnken Design for Ga leaching

Factors	4
Replicates	1
Base runs	27
Base blocks	1
Total runs	33

Table S5. Implemented parameters and levels in the Box-Behnken Design study

**Factors**	**Units**	**Symbol**	**Level low (-1)**	**Level mid (0)**	Level high (+1)
Concentration	mM	A	100	550	1000
Pulp Density	g/L	B	10	30	50
Temperature	°C	C	30	60	90
Time	h	D	3	5	7

Table S6. Complementary replicates around the center points

**Sample**	**A**	**B**	**C**	**D**	**Response**
#1	550	30	60	5	7.66
#2	550	30	60	5	4.6
#3	550	30	60	5	1.72
#4	550	30	60	5	8.35
#5	550	30	60	5	3.93
#6	550	30	60	5	7.94

Table S7. Designated BBD experiments for Ga extraction

Run	Concentration (mM)	Pulp Density (g/L)	Temperature (°C)	Time (h)	Ga Extracted (g/L)
27	550	30	60	5	3.37
18	1000	30	30	5	0.13
8	550	30	90	7	17.41
15	550	10	90	5	5.91
13	550	10	30	5	0.00
14	550	50	30	5	0.08
2	1000	10	60	5	0.25
20	1000	30	90	5	20.21
5	550	30	30	3	0.10
26	550	30	60	5	3.45
9	100	30	60	3	0.17
1	100	10	60	5	1.41
4	1000	50	60	5	6.10
21	550	10	60	3	0.57
23	550	10	60	7	2.42
17	100	30	30	5	0.01
7	550	30	30	7	0.39
22	550	50	60	3	3.24
25	550	30	60	5	2.85
6	550	30	90	3	15.23
12	1000	30	60	7	8.02
3	100	50	60	5	2.82
11	100	30	60	7	3.37
19	100	30	90	5	3.64
16	550	50	90	5	23.14
10	1000	30	60	3	1.85
24	550	50	60	7	11.64

Table S8. ANOVA summary of the quadratic model of the BBD experiments for optimizing Ga extraction

Term	Coefficient	SE	t	p-value
Intercept	4.8647	0.813	5.985	1.16E-05
A	0.0047	0.002	2.977	0.008
B	0.1519	0.035	4.316	0
C	0.2356	0.023	10.042	0
D	0.9199	0.352	2.614	0.018
A**2	-8.76E-06	4.37E-06	-2.002	0.061
B**2	-0.0014	0.002	-0.648	0.525
C**2	0.0033	0.001	3.4	0.003
D**2	0.0701	0.221	0.317	0.755
AxB	0.0001	0	0.91	0.375
AxC	0.0003	9.03E-05	3.372	0.003
AxD	0.0008	0.001	0.608	0.551
BxC	0.0071	0.002	3.516	0.002
BxD	0.0409	0.03	1.342	0.196
CxD	0.0079	0.02	0.39	0.701

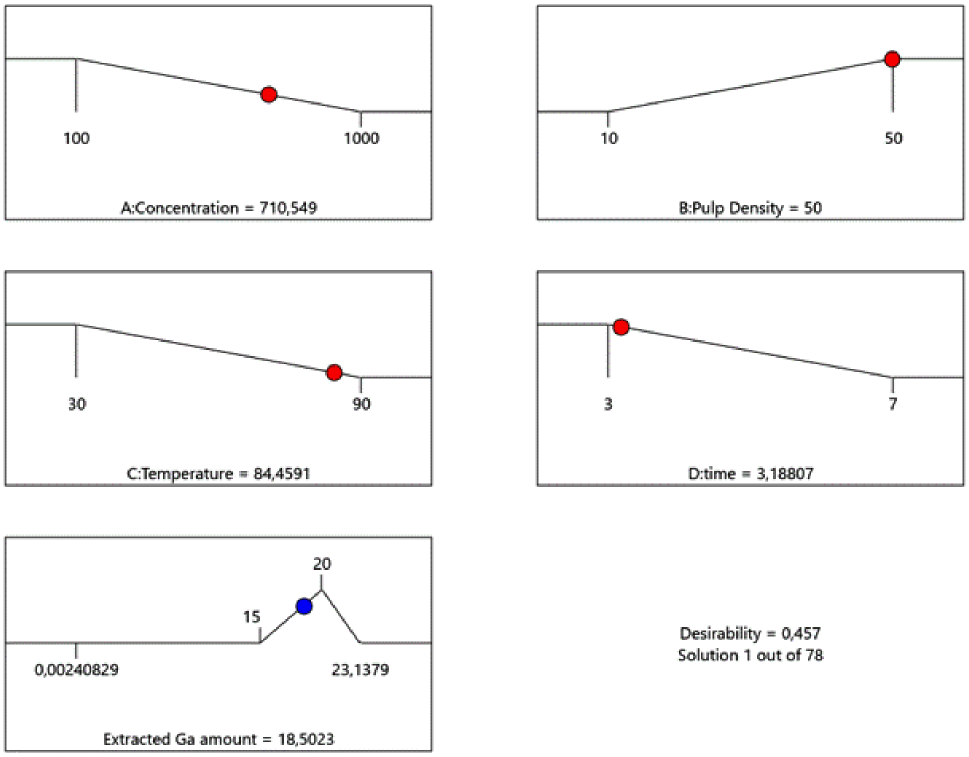

Figure S3. Desirability index for evaluationg best-possible scenarious at the lowest possible concentration, temperature, time and highest possible pulp density for maximizing Ga extraction
